# Supplementary material for: SEI growth on Lithium metal anodes in solid-state batteries quantified with coulometric titration time analysis
Source: Nat Commun. 2023 Oct 31;14:6946. doi: 10.1038/s41467-023-42512-y (PMC10618476; doi:10.1038/s41467-023-42512-y)
Supplement: Supplementary file 1 — Supplementary Information [file 41467_2023_42512_MOESM1_ESM.pdf]

SEI Growth on Lithium Metal Anodes in Solid-State Batteries Quantified with Coulometric Titration Time Analysis

Burak Aktekin[1]*, Luise M. Riegger[1], Svenja-K. Otto[1], Till Fuchs[1], Anja Henss[1], Jürgen Janek[1]*

[1]*Institute of Physical Chemistry & Center for Materials Research, Justus-Liebig-Universität Giessen, D-35392 Giessen, Germany*

**burak.aktekin@phys.chemie.uni-giessen.de, *juergen.janek@phys.chemie.uni-giessen.de*

Supplementary Information

Supporting Table 1. Estimation of number of moles of Li_6PS_5Cl and lithium metal reacted during each titration step (1 µAh) in standard CTTA experiments.

Supporting Figure 1. Internal cell resistance trends during high-current CTTA experiments.

Supporting Figure 2. Effect of temperature in CTTA experiments.

Supporting Figure 3. In situ Li deposition on stainless steel foil.

Supporting Figure 4. CTTA results of Cu | LLZO | Li pouch cells and Cu | LLZO | LPSCl | Li hybrid press cells.

Supporting Figure 5. Fractured spherical particles on CC during sample preparation.

Supporting Figure 6. Additional SEM images showing the heterogeneous parts of the stainless-steel collectors.

Supporting Figure 7. CTTA results of a cell which failed due to short-circuit.

Supporting Figure 8. XPS results after 40 µAh charge accumulation.

Supporting Figure 9. XPS (Ar^+ sputtering) results after 40 µAh charge accumulation**.**

Supporting Figure 10. XPS results before and after Ar^+ sputtering.

Supporting Figure 11. XPS results after external short-circuiting experiment.

Supporting Figure 12. Supplementary ToF-SIMS depth profiling results.

Supporting Table 1. Estimation of number of moles of Li_6PS_5Cl and lithium metal reacted during each titration step (1 µAh) in standard CTTA experiments. Thickness estimations for each reactant and product are also given for 1 µAh lithium titration steps. Faraday´s constant is taken as 26800 mAh, and density values were retrieved from the Materials Project.[1] It should be noted that these estimations assume i) the reactants and reaction products given in the table, ii) consumption of titrated lithium completely in the given reaction, iii) formation of a dense and homogeneous SEI film consisting of given reaction products in the table. As shown in the manuscript via electrochemical experiments and post mortem characterization of SEI-films, additional side reactions can also make a minor contribution to the overall lithium consumption (reaction of lithium with contaminations in/on cell components or thin native oxide film on the current collector, or the current collector itself, etc.), porous and heterogeneous SEI-films can form, and compounds not given in the proposed reaction can be present in the SEI-films such as Li_2O or LiOH. Due to these reasons, SEI thickness estimates should only be used to get an approximate information on the length-scale of the SEI-films forming after a given time. We assume that these approximate thicknesses are quite reliable in most cases, if the current collector is chosen well and if samples are prepared properly.

-	n (mol)	Compound	n (mol)	Comp.	→	n (mol)	Comp.	n (mol)	Comp.	n (mol)	Comp.
**Reaction**	**1**	**Li_6PS_5Cl**	**8**	**Li**	→	**5**	**Li_2S**	**1**	**LiCl**	**1**	**Li_3P**
Density (g cm^{-3})		1.64		0.56	→		1.67		2.14		1.48
Molar mass (g mol^{-1})		268.4		6.9	→		46		42.4		51.8
Molar vol. (cm^3 mol^{-1})		163.7		12.3	→		27.5		19.8		35
n * Molar vol.		163.7		98.6	→		137.7		19.8		35
**1 µAh charge (e.g. Li^+)**	**4.7 * 10^{-9} mol of**	**Li_6PS_5Cl**	**3.73 * 10^{-8} mol of**	**Li**	→	**2.3 * 10^{-8} mol of**	**Li_2S**	**4.7 * 10^{-9} mol of**	**LiCl**	**4.7 * 10^{-9} mol of**	**Li_3P**
Volume (cm^3 $µAh^{-1}$)		7.6 * 10^{-7}		4.6 * 10^{-7}	→		6.4 * 10^{-7}		9.2 * 10^{-8}		1.6 * 10^{-7}
**Thickness (nm $µAh^{-1}$)**		**7.6**		**4.6**	→		**6.4**		**0.9**	**1.6**	
Thickness (nm $µAh^{-1}$)	12.2				→	9.0					

Ref.[1] The Materials Project: A materials genome approach to accelerating materials innovation, Anubhav Jain, Shyue Ping Ong, Geoffroy Hautier, Wei Chen, William Davidson Richards, Stephen Dacek, Shreyas Cholia, Dan Gunter, David Skinner, Gerbrand Ceder, and Kristin A. Persson.

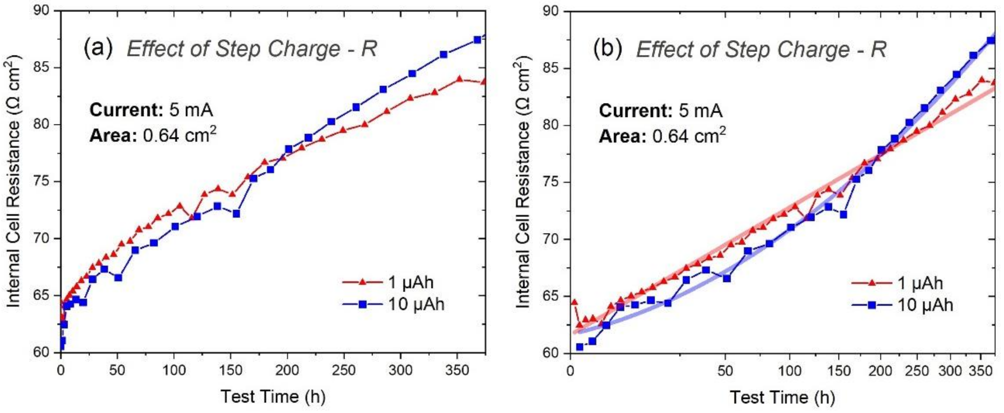

Supporting Figure 1. Internal cell resistance trends during high-current CTTA experiments. (a) Effect of step charge on internal cell resistance (calculated from the cell voltage drop before and after the end of titration step. **(b)** The data in (a) is shown with x-axis given in square-root scale.

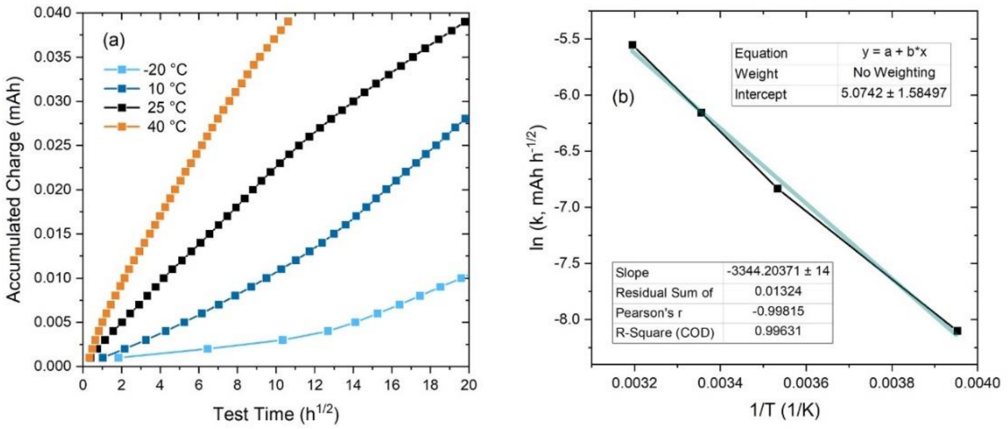

Supporting Figure 2. Effect of temperature in CTTA experiments. (a) CTTA results obtained from stainless steel **|** LPSCl **|** Li cells at different temperatures. It is seen that the linear dependency of charge from time$^{1/2}$ is not kept at lower temperatures. At 10 °C, an increase in the slope is observed around 150-200 hours and at -20 °C around 110-150 hours. **(b)** An Arrhenius-type plot is shown with the natural logarithm of the slopes in (a) plotted in *y*-axis *vs.* 1/T in the *x*-axis. For the low temperature data, e.g. 10 °C and -20 °C, the slopes of the initially linear parts were used since the increased slope observed at later regions could be a result of morphological effects (e.g. Li plating in or on the SEI).

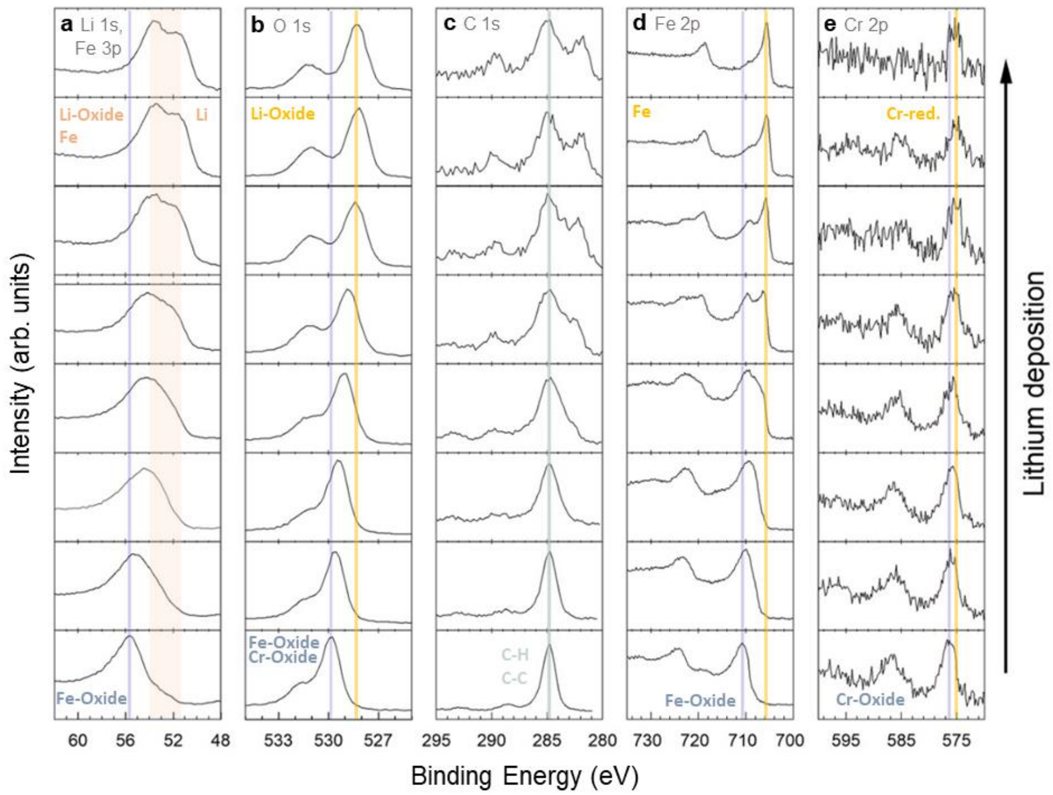

Supporting Figure 3. In situ Li deposition on stainless steel foil. The XPS results for the pristine stainless steel foil used as CC in CTTA experiments. The results of **(a)** *Li 1s / Fe 3p*, **(b)** *O 1s*, **(c)** *C 1s*, **(d)** *Fe 2p* and **(e)** *Cr 2p* spectra show that the native metal oxide film (e.g. Fe_2O_3, Cr_2O_3) on the stainless steel foil is reduced to metal phases (e.g. Fe) accompanied by the formation of Li_2O upon Li deposition.

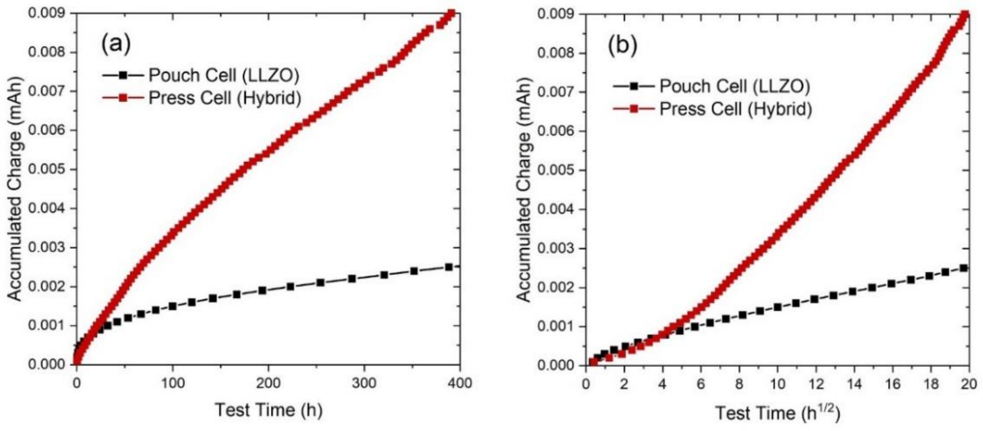

Supporting Figure 4. CTTA results of Cu | LLZO | Li pouch cells and Cu | LLZO | LPSCl | Li hybrid press cells. The accumulated charge is shown with respect to time in **(a)** and with respect to square root of time in **(b).** In pouch cells, a 100 nm Cu film was thermally deposited onto sintered LLZO pellets (the electrode area is 0.283 cm^2). In the conventional press cell, LLZO powders were pressed onto Cu foil (20 µm thick) and then pressed for a second time after LPSCl loading on top of the pressed LLZO layer. Here, LPSCl was added to act as a separator and to ensure good ionic contact with the counter electrode (Li metal) since the use of thick layer of LLZO pressed powders (without high temperature sintering) would result in high resistance.

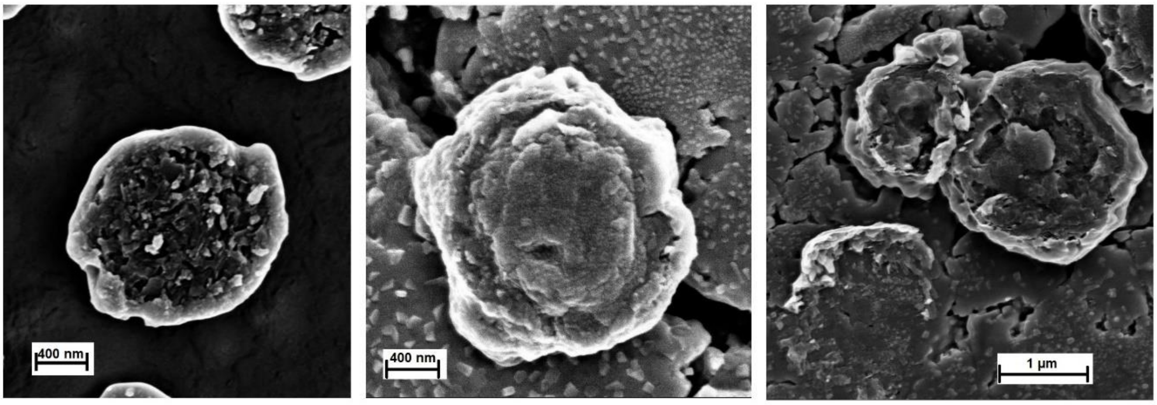

Supporting Figure 5. Fractured spherical particles on CC during sample preparation. SEM images showing the spherical particles which were fractured during the sample preparation (e.g. during the removal of stainless steel current collector from the LPSCl pellets). Cells were opened in a glovebox and sample preparation was performed directly afterwards.

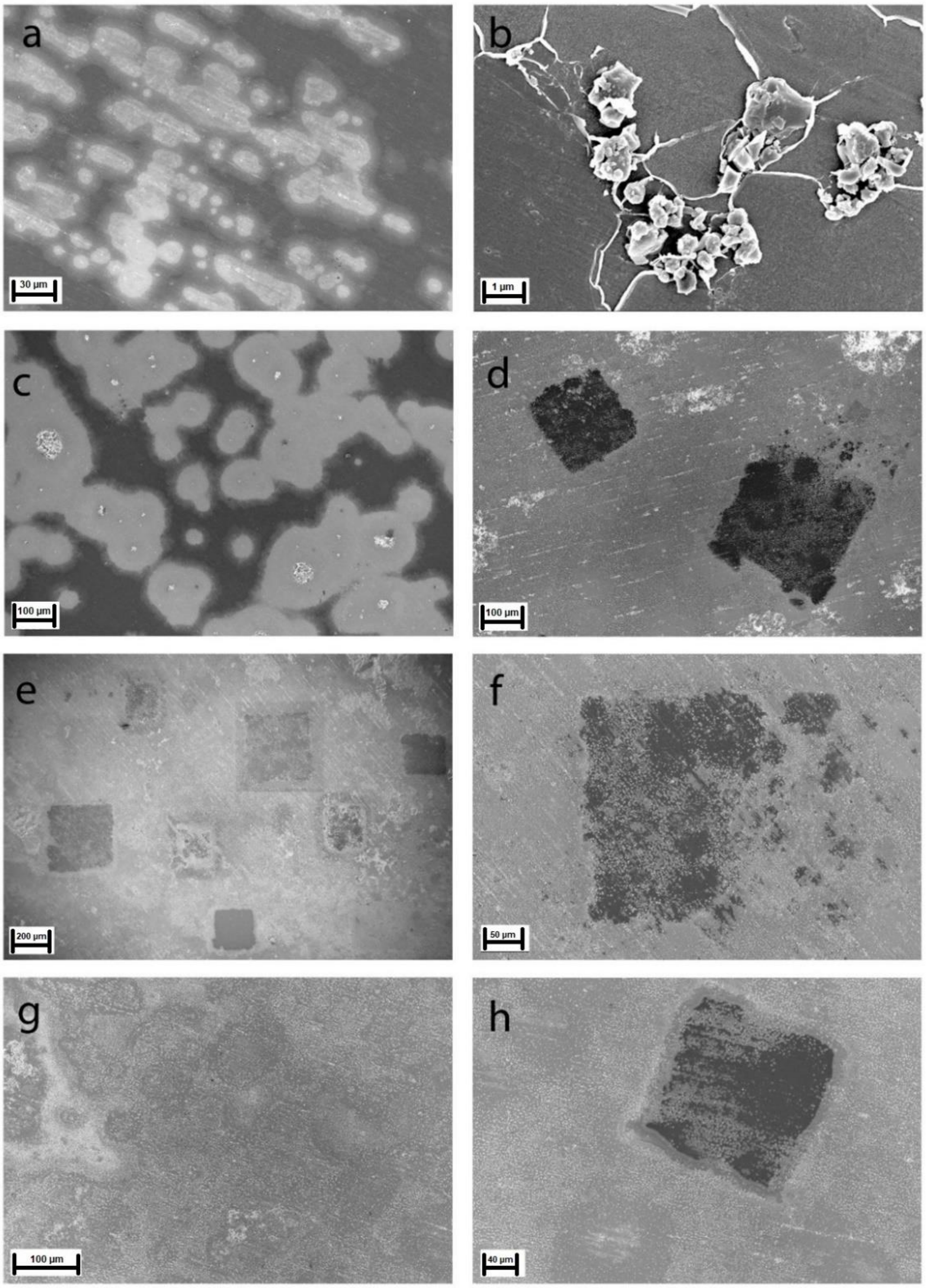

Supporting Figure 6. Additional SEM images showing the heterogeneous parts of the stainless-steel collectors. (a-b) Electrode surface after 2 µAh accumulated charge. **(c-d)** Electrode surface after 10 µAh accumulated charge. **(e-f)** Electrode surface after 40 µAh accumulated charge. **(g-h)** Electrode surface after 400 hours of short circuit storage via an external electrical cable between two electrodes. Square or rectangular shaped GCIB sputtered craters are seen in some images. Images from such areas were chosen to show the contrast of bare current collector as well as the heterogeneities through the sputtered regions. Note that the crater in **(f)** is in wedge geometry (e.g. the depth of the crater gradually increases from right edge to the left edge of the rectangle), however, some heterogeneity through the vertical axis is still observable.

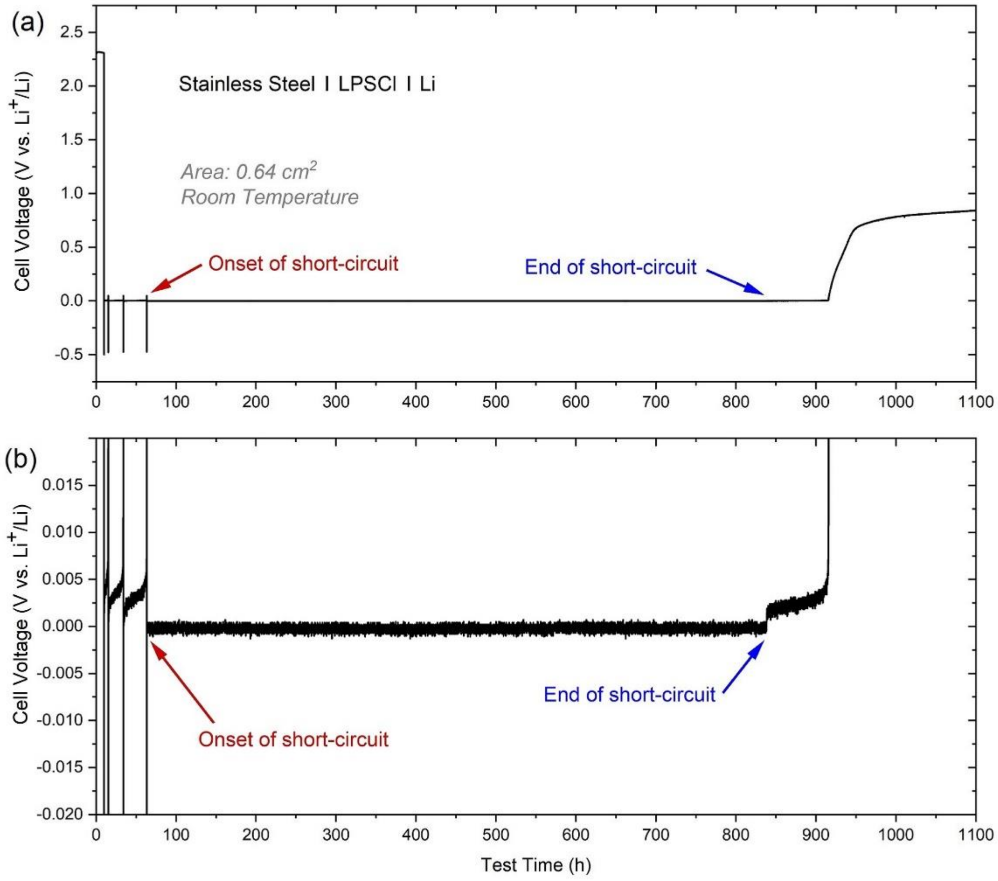

Supporting Figure 7. CTTA results of a cell which failed due to short-circuit. (a) CTTA results of a stainless steel ❙ LPSCl ❙ Li cell (25 °C, 10 µAh charge steps, 5 mA current) which failed during the 4th titration step (e.g. after 40 µAh Li deposition). In **(b)**, the same results are shown with a zoomed-in *y*-axis scale near 0 V vs. Li^+/Li. Even though the short-circuit occurs, the side reactions around the Li dendrites are expected to continue consuming Li metal. This would eventually self-heal the short-circuit pathway. Interestingly, the time required for self-healing is significantly higher as compared to expected time duration for the complete consumption of titrated Li for this cell geometry/configuration. This is likely a result of achieving electrical contact between the Li dendrites and the Li metal counter electrode. The Li metal counter electrode has a large excess of Li inventory and a 0.64 cm^2 surface area on which an extensive SEI growth already occurred prior to short-circuit. Upon the electrical contact of dendrites with the Li metal counter electrode (i.e. onset of internal short-circuit), some portion of Li required for the SEI growth could be provided from the counter electrode and thus the Li dissolution rate of dendrites would be slowed down. This would explain the observed long durations for the self-healing of Li dendrite short-circuits.

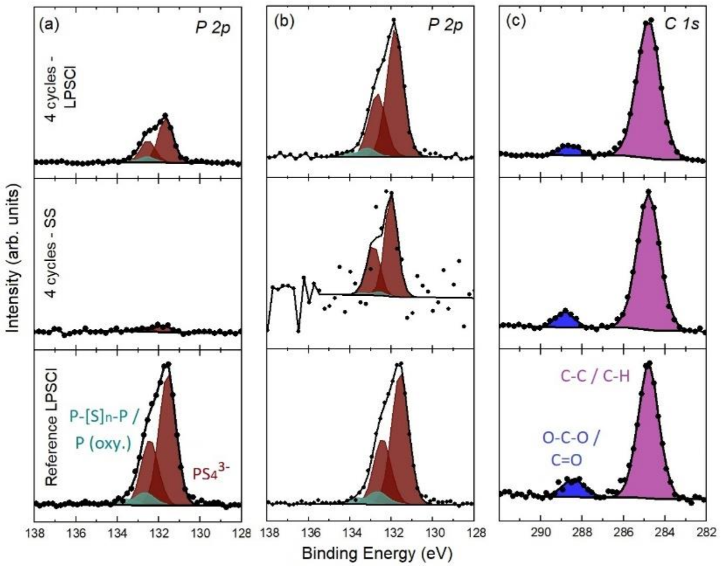

Supporting Figure 8. XPS results after 40 µAh charge accumulation. P 2p and C 1s XPS spectra of reference LPSCl electrolyte, the stainless steel current collector after the CTTA experiment (nearly 400 hours, and 40 µAh charge accumulation), and the LPSCl pellet-side facing this current collector. In **(a)**, P 2p spectra of different samples are shown as normalized to their maximum C 1s intensities. In **(b)** and **(c)**, spectra are shown with individual intensity normalization of each spectrum (divided by the maximum intensity).

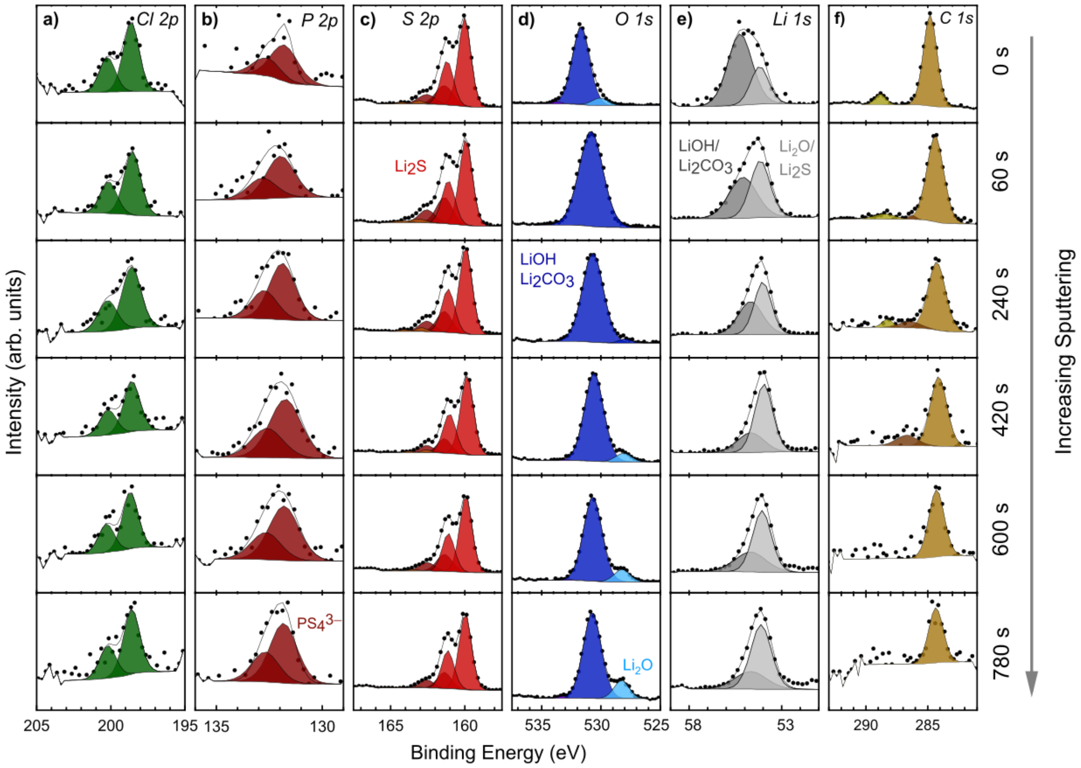

Supporting Figure 9. XPS (Ar^+ sputtering) results after 40 µAh charge accumulation. XPS (Ar^+ sputtering) results of current collector (stainless-steel) after the CTTA experiment (nearly 400 hours, and 40 µAh charge accumulation). Spectra before sputtering were energy calibrated with respect to the hydrocarbon peak positioned at E_B(C 1s) = 284.8 eV. However, the spectra measured after Ar^+ sputtering were energy calibrated following the S 2p peaks (representing SEI; electronically non-conductive part of the sample) whose binding energies were determined before the sputtering (i.e. 0 s) with E_B(C 1s) = 284.8 eV energy calibration. All spectra are shown with individual intensity normalization of each spectrum (divided by the maximum intensity).

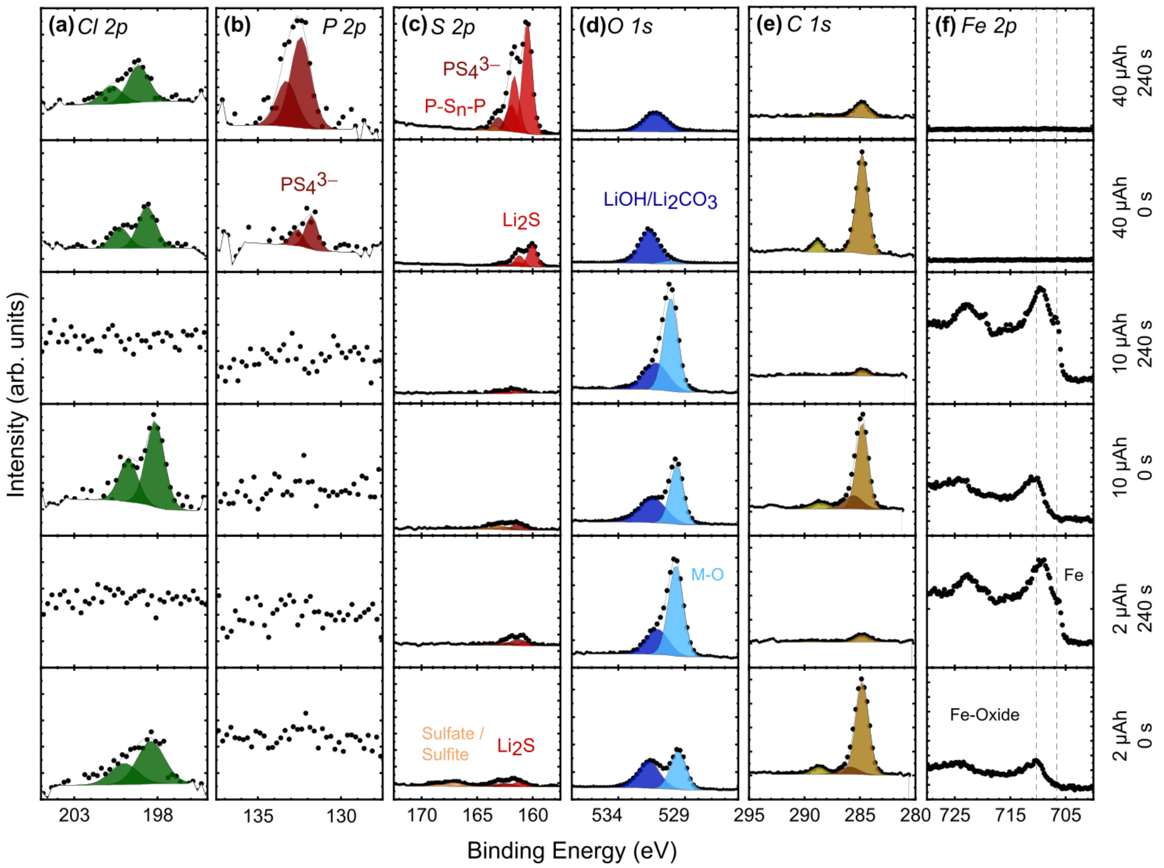

Supporting Figure 10. XPS results before and after Ar+ sputtering. XPS results (before and after sputtering) of **(a)** *Cl 2p*, **(b)** *P 2p*, **(c)** *S 2p*, **(d)** *O 1s*, **(e)** *C 1s* and **(f)** *Fe 2p* core levels for the CTTA samples (stainless-steel CC-side) tested for different durations which resulted in 2 µAh, 10 µAh and 40 µAh charge accumulation. All spectra, including the spectra measured after sputtering, were energy calibrated with respect to the hydrocarbon peak positioned at E_B(C 1s) = 284.8 eV (differential charging effects are observed after sputtering the samples, therefore, calibration for all spectra were performed in the same way to ensure more consistent interpretation of binding energies related to current collector). The C 1s spectrum of each sample (before sputtering) was used for the intensity normalization (note that each element column has its own y-scaling and therefore intensities in the graph should only be compared within each element column, and not between different element columns).

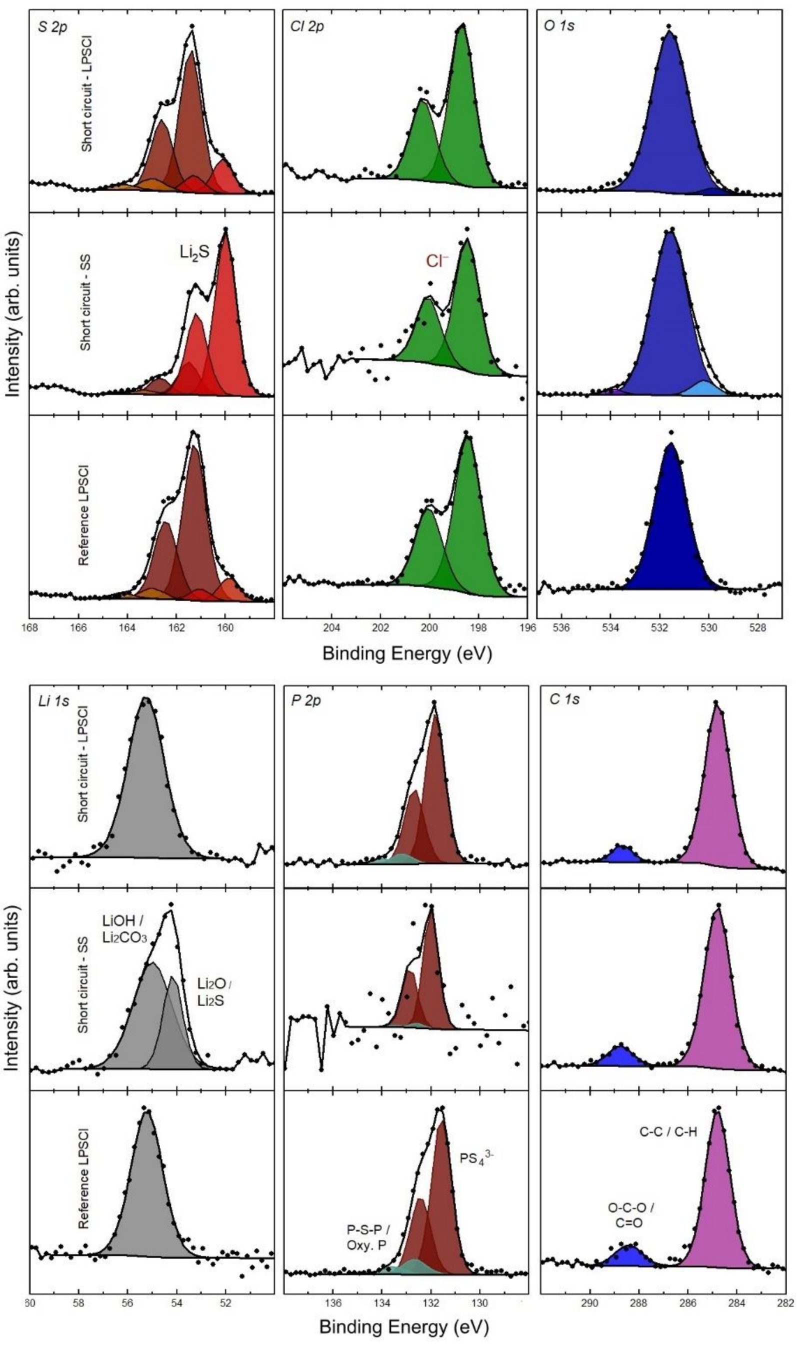

Supporting Figure 11. XPS results after external short-circuiting experiment. XPS results of current collector and LPSCl side of cells after the external short-circuit experiment (for 400 hours). The general trends are similar to the CTTA sample tested for the same duration indicating that the effect of direct Li metal deposition on the SEI chemistry is limited. All spectra were energy calibrated with respect to the hydrocarbon peak positioned at E_B(C 1s) = 284.8 eV. All spectra are shown with individual intensity normalization of each spectrum (divided by the maximum intensity).

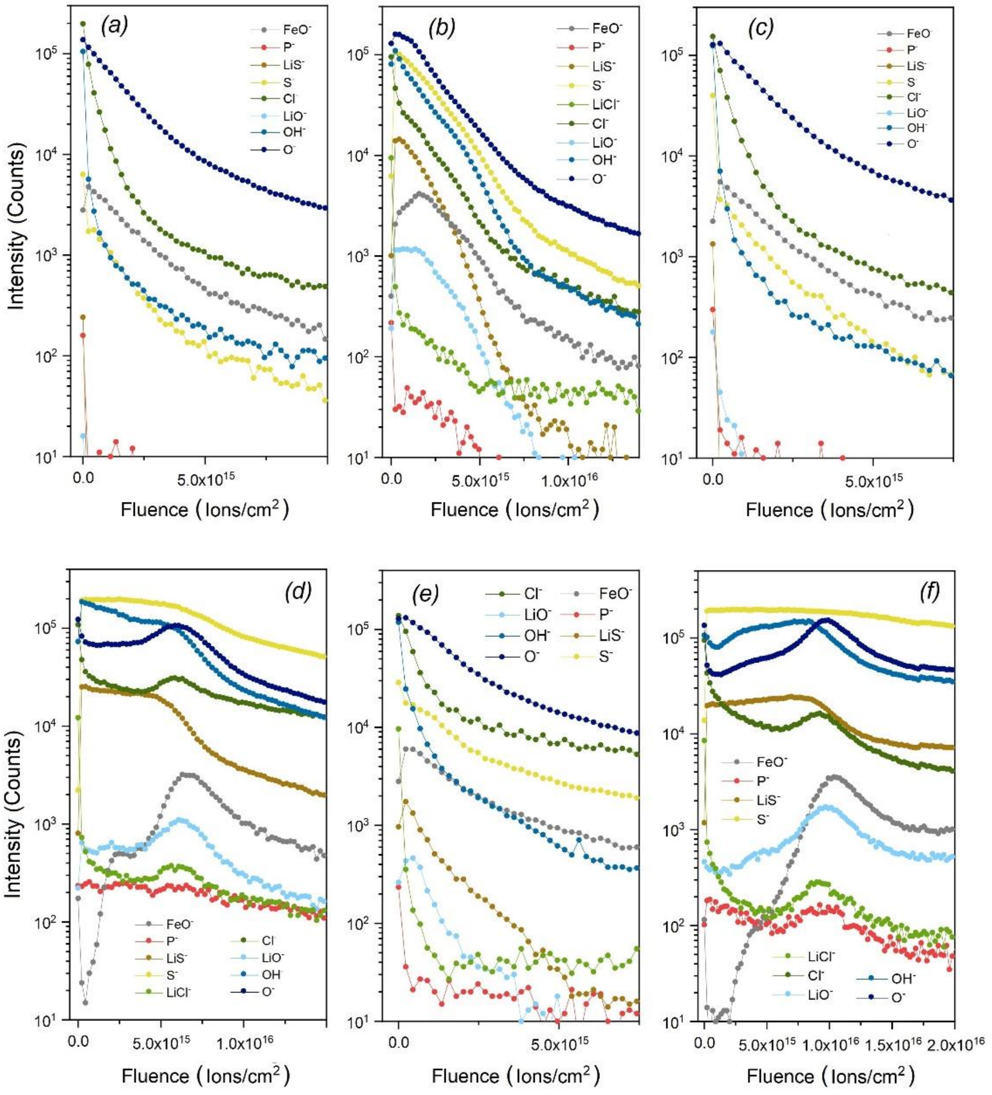

Supporting Figure 12. Supplementary ToF-SIMS depth profiling results. (a) Stainless-steel current collector (reference), **(b,c)** Two representative spots showing the SEI-rich and SEI-deficient regions for the 2 μAh sample and **(d, e)** for the 10 μAh sample. **(f)** Short-circuited sample (400 hours).
